# Plasma metabolites in patients of the REDINSCOR III registry hospitalised for de novo heart failure with preserved ejection fraction: Prognostic implications of linoleamide and 2‐trans,4‐cisdecadienoylcarnitine levels

**DOI:** 10.1002/ctm2.70398

**Published:** 2025-09-14

**Authors:** Marta Delgado‐Arija, M Dolores García‐Cosío Carmena, Manuel Martínez‐Sellés, José M Guerra, Sandra Valdivielso, José R González‐Juanatey, Mercedes Rivas‐Lasarte, Esther Roselló‐Lletí, Julián Pérez‐Villacastín, Anna Carrasquer, Lucía Matute‐Blanco, Antonio Grande‐Trillo, Maria Generosa Crespo‐Leiro, Juan F Delgado, Luis Martínez‐Dolz, REDINSCOR III registry

**Affiliations:** ^1^ Clinical and Translational Research in Cardiology Unit, Health Research Institute Hospital La Fe (IIS La Fe) Valencia Spain; ^2^ Center for Biomedical Research Network on Cardiovascular Diseases (CIBERCV), Instituto de Salud Carlos III Madrid Spain; ^3^ Cardiology Department Hospital 12 de Octubre, Instituto de Investigación Sanitaria Hospital 12 de Octubre (imas12) Madrid Spain; ^4^ Cardiology Department Hospital General Universitario Gregorio Marañón, Instituto de Investigación Sanitaria Gregorio Marañón, Universidad Europea, Universidad Complutense Madrid Spain; ^5^ Cardiology Department Hospital de la Santa Creu i Sant Pau, IR SANT PAU, Universitat Autònoma de Barcelona Barcelona Spain; ^6^ Cardiology Department Hospital del Mar, Hospital del Mar Research Institute Barcelona Spain; ^7^ Cardiology Department University Hospital, IDIS Santiago de Compostela Spain; ^8^ Cardiology Department Unidad de Insuficiencia Cardiaca Avanzada, Hospital Universitario Puerta de Hierro Majadahonda Madrid Spain; ^9^ Cardiology Department Hospital Clínico San Carlos Madrid Spain; ^10^ Cardiology Department Hospital Universitario Joan XXIII de Tarragona (Spain), IISPV, Universidad Rovira Virgili Tarragona Spain; ^11^ Cardiology Department Hospital Universitari Arnau de Vilanova, Institut Català de la Salut. IRBLleida Lleida Spain; ^12^ Cardiology Department Unidad de IC avanzada y Trasplante Cardíaco, Hospital Universitario Virgen del Rocío Sevilla Spain; ^13^ Department of Cardiology Complexo Hospitalario Universitario a Coruña (CHUAC), Instituto de Investigación Biomédica a Coruña (INIBIC), Universidad de A Coruña (UDC) A Coruña Spain; ^14^ Heart Failure and Transplantation Unit, Cardiology Department, University and Polytechnic La Fe Hospital Valencia Spain

1

Dear Editor

Heart failure with preserved ejection fraction (HFpEF) constitutes approximately 50% of all heart failure (HF) diagnoses. Despite its prevalence, effective therapies are limited given the incomplete understanding of its pathogenesis and pathophysiology.^1^ Taking into account these considerations, in this multicentre cohort of new‐onset HFpEF hospitalised patients, we described a comprehensive untargeted metabolomic study to determine endogenous metabolites that were associated with the risk of suffering an event of death or readmission for cardiovascular causes.

A total of 126 plasma samples were analysed (Figure S1), including those from 105 patients with new‐onset HFpEF. At enrolment, the median age of these patients was 75 ± 10 years, 51% were male, and in the previous year mostly were pauci‐symptomatic (81% NYHA class I‐II). At the last evaluation, 18 patients suffered an event (all‐cause mortality or cardiovascular readmission), while 82 patients did not. Table 1 summarises the demographic and clinical characteristics of HFpEF patients by event status at the end of the follow‐up. Both groups were similar regarding variables except for haemoglobin, creatinine and NT‐proBNP values prior to discharge. Additionally, the 20 healthy controls (CNT) individuals had a mean age of 66 ± 5 years, and 60% were male.

**TABLE 1:** Baseline characteristics of enrolled HFpEF patients.

	Overall (*n* = 105)	No event (*n* = 82)	Event (*n* = 18)	*p* Value
**Demographic data**				
Age (years)	75±10	75 ± 10	76 ± 11	.691
Male sex (%)	51	52	50	1.000
Body mass index (kg/m^2^)	31±7.9	30 ± 6.6	34 ± 11	.267
Current smoker (%)	15	15	17	.713
**Previous year NYHA classification (%)**				
I	39	40	35	.792
II	42	41	47	.788
III	17	19	12	.729
IV	1.9	1.2	5.9	.318
**Comorbidities**				
Hypertension (%)	70	68	83	.260
Diabetes mellitus (%)	28	27	39	.762
Dyslipidemia (%)	50	56	39	.204
Paroxysmal atrial fibrillation (%)	16	17	17	1.000
Permanent atrial fibrillation (%)	19	18	17	1.000
Previous ischemic heart disease (%)	8.6	7.3	11	.632
**Echo‐Doppler study**				
Ejection fraction (%)	59 ± 6.8	59 ± 7.2	61 ± 4.5	.112
Valve disease (moderate or severe) (%)	13	12	22	.261
**Laboratory data**				
Haemoglobin (g/dL)	129 ± 20	130 ± 18	118 ± 25	.016
Creatinine (mg/dL)	1.2 ± 0.5	1.1 ± 0.4	1.6 ± 0.8	.012
NT‐proBNP prior to discharge (pg/mL)	1144 (430–2101)	914 (366–1705)	1770 (1058–4159)	.008
NT‐proBNP at admission (pg/mL)	2525 (1554–4523)	2342 (1546–4142)	3383 (1566–4777)	.309

*Note*: *p* Value is the comparison between patients who suffered an event of death or readmission for cardiovascular causes and patients who did not.

Abbreviations: HFpEF, heart failure with preserved ejection fraction; NT‐proBNP, N‐terminal fragment of B‐type natriuretic peptide; NYHA, New York Heart Association.

Metabolic differences between CNT and HFpEF patients groups were assessed after data preprocessing in both positive and negative ESI modes (Figure S2). As shown in Table S1, we identified 24 metabolites that showed statistically significant differences between HFpEF patients and the CNT group, with a fold change threshold of |FC| ≥ 2. Regarding this finding, we performed an analysis between the HFpEF patients who experienced an event with those who did not to discover if there were metabolomic changes. As a result, seven metabolites exhibited significant differences, being linoleamide levels the ones that were most altered in this comparison (Table S1). Then, we focused on linoleamide, an endogenous lipid which is structurally related to sphingosine and sphiganine and closely related with sarco/endoplasmic reticulum Ca^2+^‐ATPase (SERCA) activity,^2^, ^3^ whose levels were higher in patients who were rehospitalised for an event (*p* < .0001) (Figure S3A). Previously, Tarazón et al.^4^ determined through untargeted metabolomics that circulating sphingosine‐1‐phosphate (S1P), a metabolite also closely related with SERCA, could be a novel approach to detect cardiac rejection. In addition, significant alterations in the main components of the sphingolipid metabolism pathways and an accumulation of SP1 was observed in HF patients.^5^ Collectively, these findings highlight the potential role of linoleamide in HFpEF patients who experienced an event. Furthermore, it is widely known that the heart is a mitochondrion‐rich tissue, and at the molecular level, impaired mitochondrial function has been suggested to contribute to HFpEF development.^6^ Circulating plasma acylcarnitines, which are intermediates of mitochondrial β‐oxidation of fatty acids, are an emerging molecular signature of HF and are thought to reflect mitochondrial dysfunction.^7^ Considering that it is well‐established that alterations in acylcarnitines levels are associated with HF risk,^8^, ^9^ we wanted to investigate the influence of 2‐trans,4‐cis‐decadienoylcarnitine, a medium‐chain acylcarnitine, whose levels were also higher in patients who experienced an event (*p* < .01) (Figure S3B). Previously, Zhang and Zhang^10^ reported that the inactivity of mitochondrial relevant NADP‐dependent enzymes provoked elevated plasma 2‐trans,4‐cis‐decadienoylcarnitine levels. Here, we observed an upregulation of 2‐trans,4‐cis‐decadienoylcarnitine as in previous studies, suggesting its involvement in mitochondrial dysfunction in HFpEF patients.

ROC curves were performed to analyse the capability of linoleamide and 2‐trans,4‐cis‐decadienoylcarnitine for predicting an event. After adjusting by age and gender, we obtained an AUC value of 0.726 (*p *< .01) for linoleamide and 0.710 (*p* < .01) for 2‐trans,4‐cis‐decadienoylcarnitine. The combination of both metabolites was associated with improved predictive accuracy, with an AUC value of 0.785 (*p* < .0001), which was higher than the AUC values for each metabolite alone (Figure 1 and Table S2).

**FIGURE 1:**
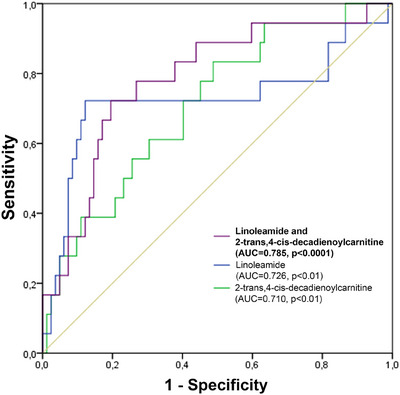
Circulating linoleamide, 2‐trans,4‐cis‐decadienoylcarnitine, and combined receiver‐operating characteristic curves for the prediction of an event of death or readmission for cardiovascular causes within 12 months of follow‐up, adjusted by age and gender. AUC, area under the curve; ROC, receiver‐operating characteristic.

Further, the relationship between metabolites and event risk was assessed using COX proportional hazard regression. Firstly, univariate tests were made to detect differences between patients with and without an event of death or readmission for cardiovascular causes, obtaining a significant influence of linoleamide, 2‐trans,4‐cis‐decadienoylcarnitine and NT‐proBNP prior to discharge (Table S3). Next, a univariate study using COX regression was proposed given that it detects the influence of linoleamide, 2‐trans,4‐cis‐decadienoylcarnitine and NT‐proBNP prior to discharge, independently, on the time until the event. Here we obtained that only both metabolites were significantly associated with the risk of suffering an event (Table 2), NT‐proBNP did not demonstrate a significant effect (*p* = .43). Considering these univariate results, a multivariate COX regression model was proposed, obtaining better AIC and C‐statistic values, suggesting enhanced prognostic value for predicting the event in HFpEF patients (Table 2).

**TABLE 2:** Association between metabolites and the risk of suffering an event.

	Univariate analysis				Multivariate analysis			
Metabolites	Hazard ratio [95% CI]	*C*‐statics [95% CI]	AIC	*p*‐value	Hazard ratio [95% CI]	*C*‐statics [95% CI]	AIC	*p*‐Value
Linoleamide	1.25 [1.11–1.41]	0.75 [0.62–0.87]	154	<.001	1.22 [1.08–1.37]			<.01
						0.77 [0.65–0.88]	151	
2‐trans,4‐cis‐ decadienoylcarnitine	2.35 [1.30–4.23]	0.67 [0.54–0.79]	156	<.01	2.12 [1.13–3.97]			<.05

Abbreviation: AIC, Akaike information criterion.

Then, from the multivariate model, a time‐dependent ROC curve was constructed to evaluate its performance in the adjusted observations. We took 3, 6 and 10 months to study the performance of the multivariate model, obtaining for 3 months an AUC = 0.761, for 6 months an AUC = 0.800 and for 10 months an AUC = 0.794 (Figure 2A and Table S4). Furthermore, a Kaplan–Meier survival model was performed stratifying patients in different groups depending on the metabolites respective median values. Significant differences were found between the survival curves (*p* < .01), in particular; between the curves of group 1 and group 4 (*p* < .05) (Figure 2B). Patients in group 4, which had levels of both metabolites above their respective medians, suffered poorer clinical outcomes than patients in group 1, who had both metabolites levels below their respective median. This finding is in line with our premise given that both metabolites were associated with an increased risk of suffering an event of death or readmission for cardiovascular causes, and both showed higher levels in patients who suffered an event compared with patients who did not.

**FIGURE 2:**
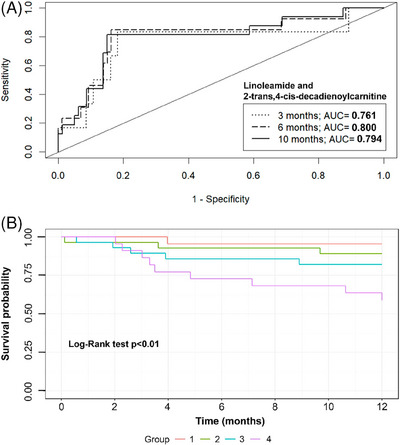
Prognostic evaluation of the multivariate model and survival analysis based on metabolite levels in HFpEF patients. Combined metabolites for time‐dependent ROC curve (A) and Kaplan–Meier curve (B). Group 1 consisted of patients with levels of both metabolites below their respective median values; group 2 included patients with 2‐trans,4‐cis‐decadienoylcarnitine levels above its median and linoleamide levels below its median; group 3 comprised patients with linoleamide levels above its median and 2‐trans,4‐cis‐decadienoylcarnitine levels below its median; and group 4 consisted of patients with levels of both metabolites above their respective medians. AUC, area under the curve; ROC, receiver‐operating characteristic.

Our study is limited on several points. The small number of clinical events may increase the risk of overfitting in the multivariate model, which should be taken into account when interpreting the results. Furthermore, although this study benefits from a multicentre design, which enhances the diversity and generalisability of the sample, it lacks validation in an entirely independent external cohort. Future research should focus on validating these biomarkers in separate patient cohorts to strengthen their clinical utility.

In conclusion, a combination of linoleamide and 2‐trans,4‐cis‐decadienoylcarnitine plasma levels could be a novel approach to determine HFpEF prognosis. This discovery contributes to progress in the knowledge of HFpEF pathophysiology, raises questions about the role of these metabolites in its diagnosis and prognosis, and opens the way for future studies of potential therapeutic targets.

## AUTHOR CONTRIBUTIONS

MDA, ERL, and LMD: designing the study, acquiring and analysing the data, contributing to data interpretation, writing the manuscript. MDGC, MMS, JMG, SV, JPV, AC, LMB, AGT, MGCL, and JFD: acquiring data and contributing to the critical review.

## CONFLICT OF INTEREST STATEMENT

The authors declare no competing interests.

## ETHICS STATEMENT

The study complied with the Declaration of Helsinki and was approved by the participating centres’ medical ethics committees. Prior to sample collection, signed informed consent was obtained from each patient.

## Supporting information



Supporting Information
