# Supplementary material for: Plasma metabolites in patients of the REDINSCOR III registry hospitalised for de novo heart failure with preserved ejection fraction: Prognostic implications of linoleamide and 2‐trans,4‐cisdecadienoylcarnitine levels
Source: Clin Transl Med. 2025 Sep 14;15(9):e70398. doi: 10.1002/ctm2.70398 (PMC12434174; doi:10.1002/ctm2.70398)
Supplement: Supplementary file 1 — Supporting Information [file CTM2-15-e70398-s001.docx]

**Supplementary Information**

**Plasma metabolites in patients of the** [**REDINSCOR III registry**](https://pubmed.ncbi.nlm.nih.gov/?term=REDINSCOR+III+registry%5BCorporate+Author%5D) **hospitalized for de novo heart failure with preserved ejection fraction: prognostic implications of linoleamide and 2-trans,4-cisdecadienoylcarnitine levels**

Marta Delgado-Arija, PhD^1,2^; M Dolores García-Cosío Carmena MD, PhD^2,3^; Manuel Martínez-Sellés MD, PhD^2,4^; José M Guerra MD, PhD^2,5^; Sandra Valdivielso, MD^6^; José R González-Juanatey, MD^2,7^; Mercedes Rivas-Lasarte MD, PhD^2,8^; Esther Roselló-Lletí, PhD^1,2^; Julián Pérez-Villacastín MD, PhD^2,9^; Anna Carrasquer, MD^10^; Lucía Matute-Blanco, MD^11^; Antonio Grande-Trillo, MD^12^; Maria Generosa Crespo-Leiro MD, PhD^2,13^; Juan F Delgado MD, PhD^2,3^; Luis Martínez-Dolz MD, PhD^1,2,14^; [REDINSCOR III registry](https://pubmed.ncbi.nlm.nih.gov/?term=REDINSCOR+III+registry%5BCorporate+Author%5D).

^1^Clinical and Translational Research in Cardiology Unit, Health Research Institute Hospital La Fe (IIS La Fe), Avd. Fernando Abril Martorell 106, 46026 Valencia, Spain. ^2^Center for Biomedical Research Network on Cardiovascular Diseases (CIBERCV), Avd. Monforte de Lemos 3-5, 28029 Madrid, Spain. ^3^Cardiology Department, Hospital 12 de Octubre, Instituto de Investigación Sanitaria Hospital 12 de Octubre (imas12), Madrid, Spain. ^4^Cardiology Department, Hospital General Universitario Gregorio Marañón, Instituto de Investigación Sanitaria Gregorio Marañón. Universidad Europea. Universidad Complutense. Madrid, Spain. ^5^Cardiology Department, Hospital de la Santa Creu i Sant Pau, IR SANT PAU, Universitat Autònoma de Barcelona, Spain. ^6^Cardiology Department, Hospital del Mar, Hospital del Mar Research Institute, Barcelona, Spain. ^7^Cardiology Department. University Hospital, IDIS. Santiago de Compostela; Spain. ^8^Cardiology Department, Unidad de Insuficiencia Cardiaca Avanzada. Hospital Universitario Puerta de Hierro Majadahonda, Madrid, Spain. ^9^Cardiology Department, Hospital Clínico San Carlos, Madrid, Spain. ^10^Cardiology Department. Hospital Universitario Joan XXIII de Tarragona (Spain), IISPV, Universidad Rovira Virgili. ^11^Cardiology Department., Hospital Universitari Arnau de Vilanova, Institut Català de la Salut. IRBLleida, Lleida, Spain. ^12^Cardiology Department. Unidad de IC avanzada y Trasplante Cardíaco. Hospital Universitario Virgen del Rocío, Sevilla, Spain. ^13^Department of Cardiology, Complexo Hospitalario Universitario a Coruña (CHUAC), Instituto de Investigación Biomédica a Coruña (INIBIC), Universidad de A Coruña (UDC), As Xubias 84, 15006 A Coruña, Spain. ^14^Heart Failure and Transplantation Unit, Cardiology Department, University and Polytechnic La Fe Hospital, Avd. Fernando Abril Martorell 106, 46026 Valencia, Spain.

Correspondence: Dr. Esther Roselló-Lletí, PhD; Clinical and Translational Research in Cardiology Unit Health Research Institute Hospital La Fe (IIS La Fe) Avd. Fernando Abril Martorell, 106. 46026 Valencia, Spain; Tel: +34 96 124 66 44. E-mail: [esther_rosello@iislafe.es](mailto:esther_rosello@iislafe.es)

**SUPPLEMENTARY FIGURES**


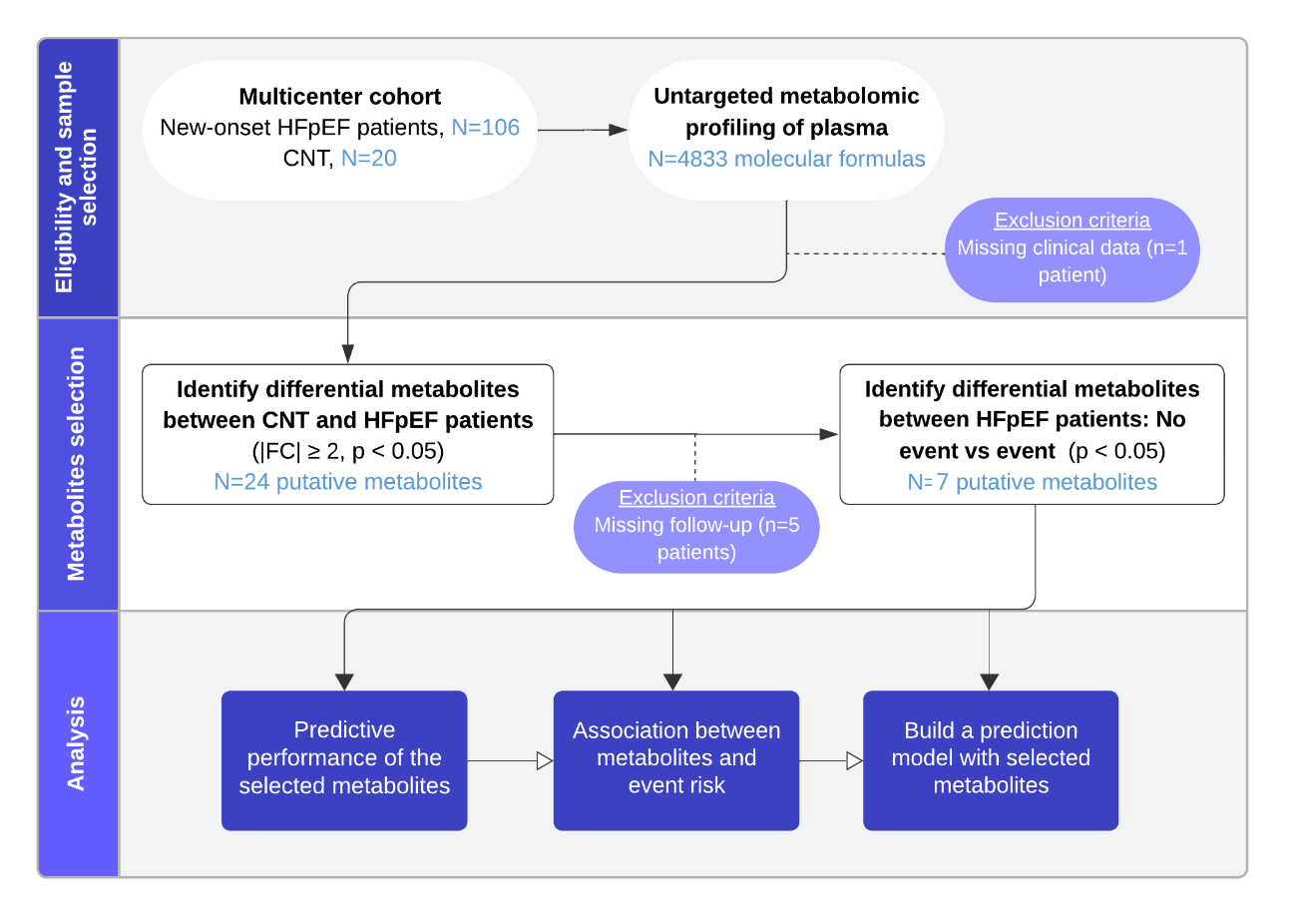
**Figure S1.** Study flowchart for sample selection, metabolites selection, and analysis process**.** CNT, control; FC, fold change; HFpEF, heart failure with preserved ejection fraction.


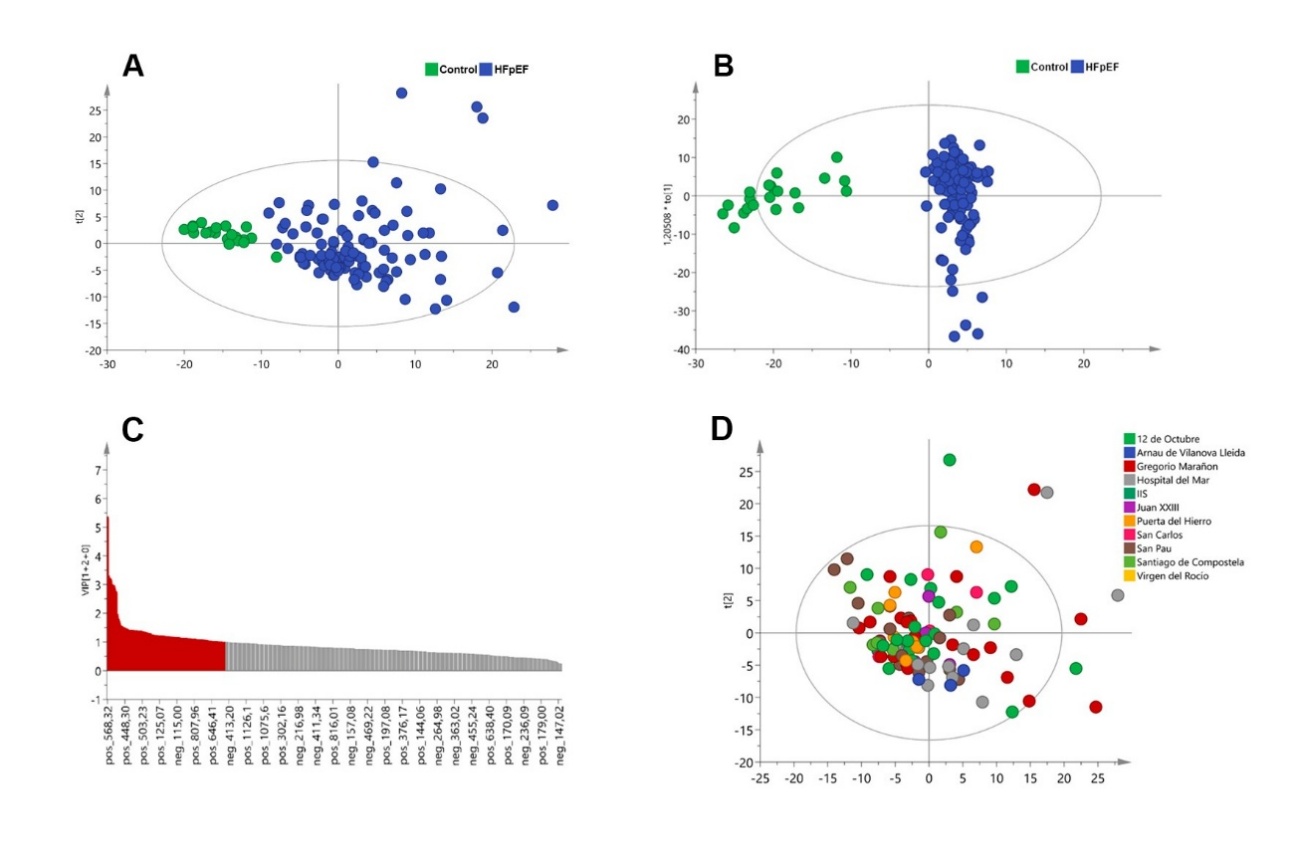
**Figure S2.** Score plots from PCA and OPLS-DA analysis**.** PCA score plot for control vs. HFpEF patients (**A**); OPLS-DA score plot for control vs. HFpEF patients (**B**); VIP >1 from control vs HFpEF patients analysis (**C**); PCA score plot for multicenter HFpEF patients (**D**). HFpEF, heart failure with preserved ejection fraction; OPLS-DA, Orthogonal-least-squares-discriminant; PCA, Principal Component Analysis; VIP, variance importance in projection.


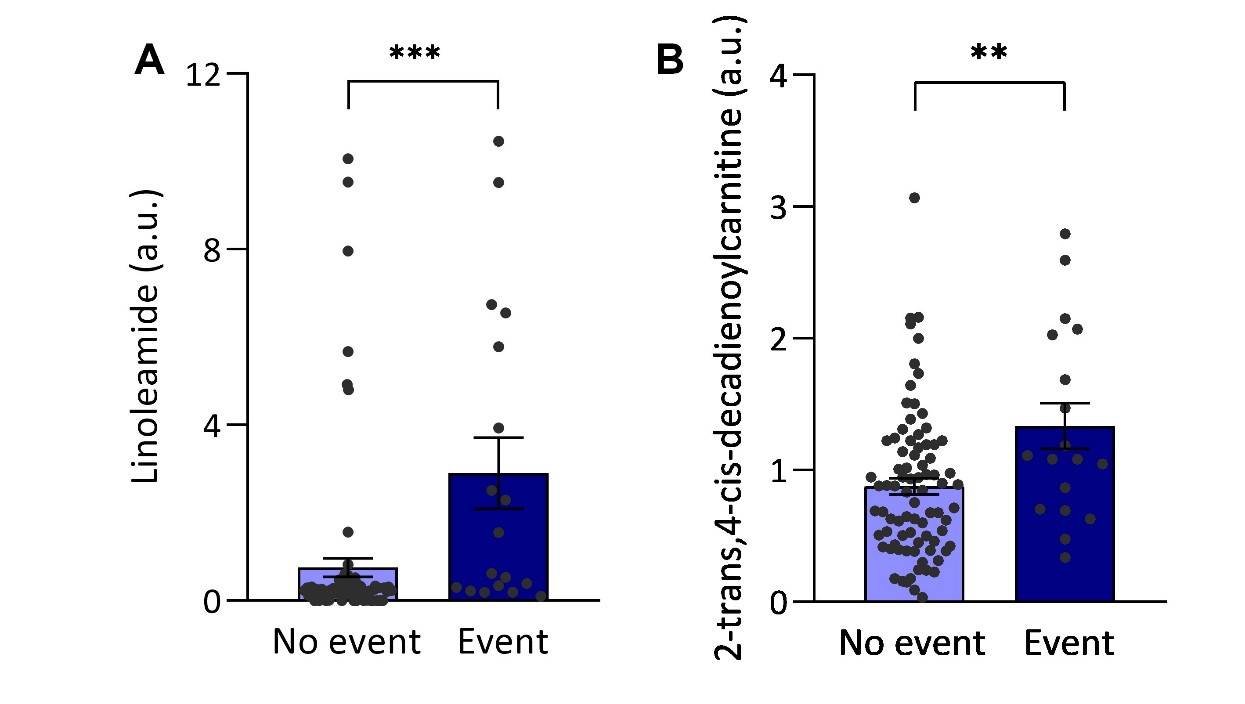
**Figure S3.** Circulating levels of linoleamide and 2-trans,4-cis-decadienoylcarnitine. Comparison of linoleamide (**A**) and 2-trans,4-cis-decadienoylcarnitine levels (**B**) between HFpEF patients who suffered an event vs who did not. **p < 0.01, *** p < 0.0001. a.u., arbitrary units; HFpEF, heart failure with preserved ejection fraction.

**SUPPLEMENTARY TABLES**

**Table S1.** Metabolites identified with different levels between the study groups (HFpEF vs CNT and HFpEF patients who experienced an event of death or readmission for cardiovascular causes with those who did not) in the metabolomics assay.

| Mass (m/z) | RT (min) | Detection mode | Formula | Adduct | SMILES | Compound name | Control vs HFpEF patients | | HFpEF patients:  No event vs event | |
| --- | --- | --- | --- | --- | --- | --- | --- | --- | --- | --- |
|  |  |  |  |  |  |  | FC | P adj | FC | P adj |
| 181.0724 | 192.8268 | + | C7H8N4O2 | M+H | CN1C2=C(NC=N2)C(=O)N(C)C1=O | Theophylline | -7.56 | <0.001 |  | NS |
|  |  |  |  |  | CN1C=NC2=C1C(=O)N(C)C(=O)N2 | Paraxanthine |  |  |  |  |
|  |  |  |  |  | CN1C=NC2=C1C(O)=NC(=O)N2C | Theobromine |  |  |  |  |
| 205.1187 | 40.9525 | + | C8H16N2O4 | M+H | CC(=O)N(O)CCCC[C@H](N)C(=O)O | N6-Acetyl-N6-hydroxy-L-lysine | -4.93 | <0.0001 |  | NS |
|  |  |  |  |  | NC(=O)CCN1CC(O)C(O)C1CO | 3,4-Dihydroxy-2-hydroxymethyl-1-pyrrolidinepropanamide |  |  |  |  |
|  |  |  |  |  | CC(C)[C@H](NC(=O)[C@@H](N)CO)C(O)=O | Serylvaline |  |  |  |  |
| 263.1242 | 42.5819 | + | C10H18N2O6 | M+H | NCCCC(NC(CCC(O)=O)C(O)=O)C(O)=O | Nopalinic acid | -2.69 | <0.0001 |  | NS |
| 280.2638 | 570.0828 | + | C18H33NO | M+H | [H]\C(CCCCC)=C(/[H])C\C([H])=C(\[H])CCCCCCCC(O)=N | Linoleamide | 3.97 | <0.001 | 3.83 | <0.0001 |
| 308.0926 | 55.5793 | + | C10H17N3O6S | M+H | N[C@@H](CCC(=O)N[C@@H](CS)C(=O)NCC(O)=O)C(O)=O | Glutathione | -4.65 | <0.001 |  |  |
| 312.2175 | 367.2121 | + | C17H29NO4 | M+H | CCCCC\C=C/C=C/C(=O)O[C@H](CC([O-])=O)C[N+](C)(C)C | 2-trans,4-cis-Decadienoylcarnitine | -2.61 | <0.01 | 1.52 | <0.01 |
| 412.2854 | 394.8921 | + | C26H37NO3 | M+H | CC(C)N(CC[C@H](C1=CC=CC=C1)C1=C(OC(=O)C(C)C)C=CC(CO)=C1)C(C)C | Fesoterodine | 5.65 | <0.0001 |  | NS |
| 450.3215 | 434.8921 | + | C26H43NO5 | M+H | CC(CCC(=O)NCC(O)=O)C1CCC2C3[C@H](O)CC4C[C@H](O)CC[C@]4(C)C3CC[C@]12C | Chenodeoxyglycocholic acid | 2.68 | <0.0001 |  | NS |
|  |  |  |  |  | [H][C@]12CCC3C4CC[C@H]([C@H](C)CCC(=O)NCC(O)=O)[C@@]4(C)[C@@H](O)CC3[C@@]1(C)CC[C@@H](O)C2 | Deoxycholic acid glycine conjugate |  |  |  |  |
|  |  |  |  |  | [H][C@@]1(CC[C@@]2([H])[C@]3([H])[C@H](O)C[C@]4([H])C[C@H](O)CC[C@]4(C)[C@@]3([H])CC[C@]12C)[C@H](C)CCC(=O)NCC(O)=O | Chenodeoxycholic acid glycine conjugate |  |  |  |  |
|  |  |  |  |  | [H][C@@]1(CC[C@@]2([H])[C@]3([H])[C@@H](O)C[C@]4([H])C[C@H](O)CC[C@]4(C)[C@@]3([H])CC[C@]12C)[C@H](C)CCC(=O)NCC(O)=O | Glycoursodeoxycholic acid |  |  |  |  |
| 466.3158 | 394.7315 | + | C26H43NO6 | M+H | C[C@@H](CCC(=O)NCC(O)=O)[C@@H]1CC[C@@H]2[C@@H]3[C@@H](C[C@@H](O)[C@]21C)[C@]1(C)CC[C@H](O)C[C@@H]1C[C@@H]3O | Glycocholic acid | 5.32 | <0.0001 |  | NS |
|  |  |  |  |  | CC(CCC(=O)NCC(O)=O)C1CCC2C3C(O)CC4CC(O)CCC4(C)C3CC(O)C12C | Sodium glycocholate |  |  |  |  |
|  |  |  |  |  | [H][C@@]1(CC[C@@]2([H])[C@]3([H])[C@@H](O)C[C@]4([H])C[C@H](O)CC[C@]4(C)[C@@]3([H])C[C@H](O)[C@]12C)[C@H](C)CCC(=O)NCC(O)=O | 3a,7b,12a-Trihydroxyoxocholanyl-Glycine |  |  |  |  |
| 597.3637 | 384.6976 | + | C33H48N4O6 | M+H | CCC1C(C)C(=O)NC1CC1=C(C)C(CCC(O)=O)=C(CC2=C(CCC(O)=O)C(C)=C(CC3NC(=O)C(CC)C3C)N2)N1 | L-Urobilinogen | 5.55 | <0.0001 | 1.72 | <0.05 |
| 668.2856 | 384.6677 | + | C39H41NO9 | M+H | COC1=C(C2CC(C)(C)OC3=C2C(O)=C2C=C(C(=O)OC2=C3C(C)(C)C=C)C(C)(C)C=C)C2=C(C(O)=C1)C(=O)C1=C(N2)C(OC)=C(O)C=C1 | Neoacrimarine B | 6.38 | <0.001 | 1.64 | <0.05 |
| 682.3008 | 400.4689 | + | C40H43NO9 | M+H | COC1=C(OC)C2=C(C=C1)C(=O)C1=C(N2)C(C2CC(C)(C)OC3=C2C(O)=C2C=C(C(=O)OC2=C3C(C)(C)C=C)C(C)(C)C=C)=C(OC)C=C1O | Neoacrimarine A | 4.09 | <0.001 |  | NS |
| 115.0036 | 57.0549 | - | C4H4O4 | M-H | OC(=O)\C=C/C(O)=O | Maleic acid | -37.32 | <0.0001 |  | NS |
|  |  |  |  |  | OC(=O)\C=C\C(O)=O | Fumaric acid |  |  |  |  |
| 116.0715 | 177.6638 | - | C5H11NO2 | M-H | NCCCCC(O)=O | 5-amino-pentanoic acid | 11.37 | <0.0001 | 1.88 | <0.01 |
| 133.0503 | 55.2684 | - | C5H10O4 | M-H | OC[C@H]1OC(O)C[C@@H]1O | Deoxyribose | 3.26 | <0.0001 | 2.04 | <0.01 |
|  |  |  |  |  | CC(CO)(CO)C(O)=O | 3-hydroxy-2-(hydroxymethyl)-2-methylpropanoic acid |  |  |  |  |
|  |  |  |  |  | CCC(O)C(O)C(O)=O | 2,3-dihydroxy-valeric acid |  |  |  |  |
|  |  |  |  |  | CC(C)(O)[C@@H](O)C(O)=O | (R)-2,3-Dihydroxy-isovalerate |  |  |  |  |
|  |  |  |  |  | CC(O)C(C)(O)C(O)=O | 2,3-Dihydroxy-2-methylbutanoic acid |  |  |  |  |
|  |  |  |  |  | OC[C@@H]1OC[C@@H](O)[C@@H]1O | 1-Deoxy-D-xylulose |  |  |  |  |
| 179.0571 | 195.2915 | - | C7H8N4O2 | M-H | CN1C2=C(NC=N2)C(=O)N(C)C1=O | Theophylline | -6.10 | <0.001 |  | NS |
|  |  |  |  |  | CN1C=NC2=C1C(=O)N(C)C(=O)N2 | Paraxanthine |  |  |  |  |
|  |  |  |  |  | CN1C=NC2=C1C(=O)NC(=O)N2C | Theobromine |  |  |  |  |
| 204.9822 | 167.8313 | - | C6H6O6S | M-H | OC1=C(O)C(OS(O)(=O)=O)=CC=C1 | Pyrogallol-1-O-sulphate | 3.81 | <0.0001 |  | NS |
|  |  |  |  |  | OC1=CC=CC(O)=C1OS(O)(=O)=O | Pyrogallol-2-O-sulphate |  |  |  |  |
|  |  |  |  |  | OS(=O)(=O)OCC1=CC=C(O1)C=O | 5-Sulfoxymethylfurfural |  |  |  |  |
| 324.0723 | 71.8929 | - | C14H15NO8 | M-H | O[C@H]1[C@H](O)[C@@H](OC2=CC3=C(C=CN3)C=C2O)O[C@H]([C@@H]1O)C(O)=O | Dihyroxy-1H-indole glucuronide I | 2.67 | <0.0001 |  | NS |
| 365.1351 | 137.5972 | - | C17H22N2O7 | M-H | OCC(O)C(O)C(O)C(O)C1NC(CC2=C1NC1=CC=CC=C21)C(O)=O | Tetrahydropentoxyline | 3.21 | <0.0001 | 1.80 | <0.01 |
|  |  |  |  |  | COC1=C(OC2OC(CO)C(O)C(O)C2O)C=CC(CC2=NC=CN2) | Semilepidinoside B |  |  |  |  |
| 464.3015 | 394.2032 | - | C26H43NO6 | M-H | [H][C@@]12CC[C@H]([C@H](C)CCC(=O)NCC(O)=O)[C@@]1(C)[C@@H](O)C[C@@]1([H])[C@@]2([H])[C@H](O)CC2C[C@H](O)CC[C@]12C | Glycocholic acid | 5.05 | <0.0001 |  | NS |
|  |  |  |  |  | [H][C@@]1(CC[C@@]2([H])[C@]3([H])[C@@H](O)C[C@]4([H])C[C@H](O)CC[C@]4(C)[C@@]3([H])C[C@H](O)[C@]12C)[C@H](C)CCC(=O)NCC(O)=O | 3a,7b,12a-Trihydroxyoxocholanyl-Glycine |  |  |  |  |
| 498.2889 | 410.8786 | - | C26H45NO6S | M-H | [H][C@@]12CC[C@H]([C@H](C)CCC(=O)NCCS(O)(=O)=O)[C@@]1(C)CC[C@@]1([H])[C@@]2([H])[C@@H](O)CC2C[C@H](O)CC[C@]12C | Tauroursodeoxycholic acid | 4.57 | <0.0001 |  | NS |
|  |  |  |  |  | [H][C@@]1(CC[C@@]2([H])[C@]3([H])[C@H](O)C[C@]4([H])C[C@H](O)CC[C@]4(C)[C@@]3([H])CC[C@]12C)[C@H](C)CCC(=O)NCCS(O)(=O)=O | Taurochenodeoxycholic acid |  |  |  |  |
|  |  |  |  |  | [H][C@@]1(CC[C@@]2([H])[C@]3([H])CC[C@]4([H])C[C@H](O)CC[C@]4(C)[C@@]3([H])C[C@H](O)[C@]12C)[C@H](C)CCC(=O)NCCS(O)(=O)=O | Taurodeoxycholic acid |  |  |  |  |
| 512.2686 | 421.8638 | - | C26H43NO7S | M-H | [H][C@@]1(CC[C@@]2([H])[C@]3([H])CC[C@]4([H])C[C@@H](CC[C@]4(C)[C@@]3([H])CC[C@]12C)OS(O)(=O)=O)[C@H](C)CCC(=O)NCC(O)=O | Sulfolithocholylglycine | 3.31 | <0.0001 |  | NS |
| 514.2842 | 369.2869 | - | C26H45NO7S | M-H | [H][C@]12C[C@H](O)CC[C@]1(C)C1C[C@H](O)[C@]3( | Taurallocholic acid | 8.49 | <0.001 |  | NS |
|  |  |  |  |  | [H][C@@]12C[C@H](O)CC[C@]1(C)C1C[C@H](O)[C@] | Tauroursocholic acid |  |  |  |  |
|  |  |  |  |  | C[C@@H](CCC(=O)NCCS(O)(=O)=O)[C@@H]1CC[C@@H]2[C@@H]3[C@@H](CC[C@]21C)[C@]1(C)CC[C@H](O)C[C@@H]1[C@@H](O)[C@H]3O | Tauro-b-muricholic acid |  |  |  |  |
|  |  |  |  |  | C[C@H](CCC(=O)NCCS(=O)(=O)O)[C@H]1CC[C@H]2C3[C@H](C[C@H](O)[C@@]21C)[C@@]1(C)CC[C@@H](O)C[C@H]1C[C@H]3O | Taurocholate |  |  |  |  |
| 526.2938 | 485.3579 | - | C27H46NO7P | M-H | [H][C@@](CO)(COP(O)(=O)OCCN)OC(=O)CC\C=C/C\C=C/C\C=C/C\C=C/C\C=C/CCCCC | LysoPE (22:5_0:0) | -2.86 | <0.01 |  | NS |
|  |  |  |  |  | [H][C@@](CO)(COP(O)(=O)OCCN)OC(=O)CCCCC\C=C/ |  |  |  |  |  |
|  |  |  |  |  | [H][C@@](O)(COC(=O)CC\C=C/C\C=C/C\C=C/C\C=C/C\C=C/CCCCC)COP(O)(=O)OCCN |  |  |  |  |  |
|  |  |  |  |  | [H][C@@](O)(COC(=O)CCCCC\C=C/C\C=C/C\C=C/C\C=C/C\C=C/CC)COP(O)(=O)OCCN |  |  |  |  |  |

Adj, adjusted; CNT, control; HFpEF, heart failure with preserved ejection fraction; FC, fold change; RT, retention time.

**Table S2.** ROC curve of circulating altered metabolites for predicting an event of death or readmission for cardiovascular causes within 12 months of follow up, adjusted by age and gender.

| **Metabolites** | **AUC** | **P-value** | **95% CI** | **Cutoff point** | **SS** | **SP** | **PPV** | **NPV** |
| --- | --- | --- | --- | --- | --- | --- | --- | --- |
| Linoleamide | 0.726 | <0.01 | 0.561-0.891 | 0.244 | 33 | 93 | 54 | 87 |
| 2-trans,4-cis-decadienoylcarnitine | 0.710 | <0.01 | 0.583-0.837 | 0.238 | 39 | 84 | 35 | 86 |
| Combined linoleamide and 2-trans,4-cis-decadienoylcarnitine | 0.785 | <0.0001 | 0.665-0.904 | 0.218 | 61 | 84 | 46 | 91 |

Sensitivities, Specificities and Predictive Values (%) were calculated with the cutoff point (FC≥1.5) which is expressed in arbitrary units. AUC, area under the curve; CI, confidence interval; FC, fold change; NPV, negative predictive value; PPV, positive predictive value; ROC, receiver operating characteristic; SP, specificity; SS, sensitivity.

**Table S3.** Univariate tests between HFpEF patients with and without an event.

| **Variable** | **P-value** |
| --- | --- |
| Sex (%) | 1.0000 |
| Age (years) | 0.6310 |
| **2-trans,4-cis-decadienoylcarnitine (a.u.)** | **0.0126** |
| **Linoleamide (a.u.)** | **0.0004** |
| Prior diabetes (%) | 0.3902 |
| Diabetes mellitus (%) | 0.7618 |
| Antidiabetics drugs (%) | 0.2451 |
| Hypertension (%) | 0.2599 |
| NT-proBNP prior to discharge(pg/ml) | **0.0076** |
| NT-proBNP at admission(pg/ml) | 0.3087 |
| Atrial fibrillation (%) | 1.0000 |
| Previous year NYHA classification (%) | 0.4969 |
| Right atrium area (cm^2^) | 0.6286 |
| Left atrium area (cm^2^) | 0.8373 |
| Left atrium diameter (mm) | 0.5859 |
| Right ventricular end diastolic diameter (mm) | 0.6579 |
| Left ventricular end diastolic diameter (mm) | 0.1897 |
| Left ventricular end systolic diameter (mm) | 0.6065 |
| E’ lateral (cm/s) | 0.5537 |
| Left ventricular posterior wall thickness (mm) | 0.2849 |
| Tricuspid annular plane systolic excursion (mm) | 0.2811 |
| Inferior vena cava diameter (mm) | 0.7700 |
| Left atrial volumen (mL) | 0.2798 |
| Left ventricular end-diastolic volumen 4C (mL) | 0.5932 |

a.u., arbitrary units; HFpEF, heart failure with preserved ejection fraction; NT-proBNP, N-terminal pro-B-type natriuretic peptide; NYHA, New York Heart Association.

| **Time** | **AUC** | **P-value** | **SS** | **SP** | **PPV** | **NPV** |
| --- | --- | --- | --- | --- | --- | --- |
| 3 months | 0.761 | <0.0001 | 83 | 66 | 14 | 98 |
| 6 months | 0.800 | <0.05 | 85 | 70 | 30 | 97 |
| 10 months | 0.794 | <0.0001 | 81 | 70 | 34 | 95 |

**Table S4.** Time-dependent ROC curve of the multivariate model

Sensitivities, specificities, and predictive values (%) for the performance of the multivariate model. AUC, area under the curve; NPV, negative predictive value; PPV, positive predictive value; ROC, receiver operating characteristic; SP, specificity; SS, sensitivity.

**SUPPLEMENTARY MATERIAL AND METHODS**

**Collection of samples**

This multicenter metabolomic study included 106 patients (>18 years) who were diagnosed for the first time with HFpEF when they were hospitalized in the cardiology service, and 20 control (CNT) individuals who presented a normal echo-Doppler study, electrocardiogram and hematologic and biochemical analyses. During 2018-2021 all individuals were registered in eleven hospitals in Spain as part of the REDINSCOR III project. The Spanish Network for the Study of Heart Failure III (REDINSCOR III) is a prospective registry that included adults admitted with new-onset HF in cardiology departments. Registered variables included clinical, biochemical and echocardiographic data, interventions and procedures, drugs, socio-economic data, and events during one-year follow-up. In brief, patients with previously undiagnosed HF admitted into the hospital either with acute signs and symptoms of HF, chest radiography with pulmonary congestion or pleural effusion and elevated natriuretic peptides (brain natriuretic peptide ≥100ng/L or NT-proBNP (N-terminal pro-B-type natriuretic peptide) ≥300ng/L) were enrolled. These patients were classified as new-onset HFpEF in agreement with the hospital's clinical cardiology team, based on patient evolution over the first 48 hours and focusing on clinical parameters, natriuretic peptide levels, and echocardiographic assessment showing a left ventricular ejection fraction ≥50%. The extraction of the sample was carried out once the patient was stabilized, at least 48 hours prior to hospital discharge. Blood samples were obtained at the hospital using peripheral venipuncture via a 10 mL glass vacuum extraction tube treated with 15% EDTA anticoagulant (0.12 mL) (BD Vacutainer K3E^R^; REF 368480, Becton, Dickinson and Company). Plasma tubes were separated by centrifugation (Eppendorf Model 5415R Centrifuge, Eppendorf Iberica S.L.U.) at 1500xg for 10 minutes at 4 °C, aliquoted, and immediately stored at −80 °C. Study HFpEF patients were divided into 2 groups depending on if they had suffered an event of death or readmission for cardiovascular causes or not in the follow-up; 1 patient with missing clinical data was excluded from the comparision between CNT and HFpEF patients and 5 more patients, with missing follow-up data, were excluded from the comparision between HFpEF patients (Figure S1).

For each sample the age, gender, body mass index, comorbidities, hemodynamic parameters, and other clinical characteristics at the time of the study enrollment were recorded (Table 1). These patients were subjected to a structured follow-up with 3 examinations at different times: 1 month, 6 months and 12 months after the study enrollment. At the end of the follow-up, all-cause mortality and cardiovascular readmissions were documented, in addition to the clinical variables.

**Plasma processing and analysis using UPLC-QToF-MS-based untargeted metabolomics**

150 μL of cold acetonitrile (0.1%, v/v) was added to 50 μL of each plasma sample. The mixture was vortexed, incubated for 20 min at − 20 °C, and centrifuged (13000xg for 10 min at 4 °C). After centrifugation, 10 μL of the supernatant was transferred to a 96-well plate for LC-QTOF-6550 analysis. In each sample, 90 μL of H2O (0.1% HCOOH, v/v) and 10 μL of internal standard mix solution (MIX STDI) (reserpine, leucine, enkephaline, phenylalanined5, 20 μM each one) were added. Quality control samples (QCs) were prepared by combining 10 μL from each extract. Blank samples, prepared to replace the extract with ultrapure water, were used to identify artefacts from reagents, the tube, and other materials. Finally, samples were injected randomly into the chromatographic system. To monitor the stabilities of the instrumental system and the instrumental drift, QC samples were injected at every 8th sample in each sequence, and the blank samples were performed at the end of the sequence. The metabolomic analysis was performed using an Ultra-Performance Liquid Chromatography (UPLC) system coupled to an iFunnel Q-ToF Agilent 6550 mass spectrometer (Agilent Technologies, CA, USA) with a UPLC BEH C18 (100 x 2.1 mm, 1.7 μm, Waters, Wexford, Ireland) column from Waters (Wexford, Ireland). The mobile phase was: solvent A (0.1% formic acid in water) and solvent B (0.1% formic acid in acetonitrile). The gradient elution was as follows: 98% A (0–1 min), 75% A (1–2 min), 50% A (2–3 min), and 5% A (3–14 min). A 95% mobile phase B was maintained for 3 min, and then a 0.55 min gradient was to return to the initial conditions. The flow rate was set at 400 μL/min with columned temperature maintained at 40 °C. The injection volume was 5 μL, and the autosampler was kept at 4 °C. The mass spectrometer worked in full scan from 50 to 1700 m/z for MS with a scan of 6 Hz collected both in positive (ESI+) and negative (ESI−) electrospray ionization modes. The settings of the electrospray ion source were set as follows: gas temperature: 200 °C; drying gas: 14 L/min; nebulizer: 60 psi; sheath gas temperature: 350 °C; sheath gas flow: 11 L/min. QC sample was also repeatedly analyzed under auto MS/MS (DDA, data-dependent fragmentation mode) and All-ion (DIA, data-independent fragmentation mode) which provides valuable information on the (de)protonated molecules and main fragment ions for the identification of discovered metabolites, providing an increased level of confidence in the metabolite annotations.

**Metabolomic data processing**

Raw metabolomic databases were converted to mzXML format using ProteoWizard (http:// prote owiza rd. sourc efora ge. net/). Then, data analysis was done using the LipidMS pre-processing tool (LipidMS 3.0: an R-package and a web-based tool for LC-MS/MS data processing and lipid annotation. bioRxiv, 2022. (doi.org/10.1101/2022.02.25.476005) for peak detection, noise filtering, peak alignment, and peak correspondence. A two-dimensional data matrix containing information on molecular features (retention time and m/z) and peak intensities across the samples was generated. Data quality (reproducibility, stability) was assessed using the internal standard’s stability and the QC’s coefficients of variation (CVs). Molecular features with CVs > 30% were excluded from the data matrix. Additionally, a QC-based robust locally weighted scatter plot smoothing LOESS normalization was applied to eliminate intra-batch variability due to technical differences. Data from positive and negative ionization modes were merged and filtered for statistical analysis.

**Metabolites ‘annotation**

Metabolites were first identified by database searching using the online CEU Mass Mediator which combines the results of the Human Metabolome Database (HMBD) (http:// hmdb. ca/), KEGG (http:// www. kegg. jp)80,81, Metlin (http:// metlin. scrip ps. edu/), LipidMaps (http:// www. lipid maps. org), and others databases, within a mass range accuracy of ± 5 ppm. The adducts included in the analysis were [M + H] and [M + Na] for ESI+ ionization mode and [M − H] and [M + HCOOH − H] for ESI− ionization mode.

**Statistical analysis**

Basal characteristics were expressed as mean ± standard deviation for continuous variables, and percentages for discrete variables. Results for each variable were tested for normality using the Kolmogorov-Smirnov method. Continuous variables not following a normal distribution were compared using the Mann-Whitney test, and categorical clinical variables were compared using the chi-square test. Variables with a normal distribution were compared using the Student’s t-test for continuous variables, and the Fisher’s exact test for discrete variables. We considered differentially expressed metabolites those with a *P* value (P adj) corrected by FDR ≤ 0.05 to avoid identification of false-positives. An initial exploratory analysis was conducted on metabolomic, demographic, clinical, and echocardiographic variables; univariate models were performed in order to investigate their association with the occurrence of events. Following this, univariate Cox regression analysis was performed to assess the relationship between each variable of interest and time to event. Variables that showed statistical significance in the previously mentioned univariate models (p-value < 0.05, Wald Test) were subsequently included in a multivariate Cox regression analysis. The Area Under the Curve (AUC), sensitivity, specificity, and predictive values of metabolites levels for the prediction of an event of death or readmission for cardiovascular causes was assessed by construction of receiver-operating characteristic (ROC) curves adjusted by age and gender. A p < 0.05 was considered statistically significant. The performance of the multivariate model was evaluated using time-dependent ROC curves at 3, 6, and 10 months, employing the adjusted hazard ratio (cut-off point = 1) for the calculations.

A Kaplan-Meier survival model was constructed to analyze the time to event based on a categorical variable derived from the levels of two metabolomic variables (linoleamide and 2-trans,4-cis-decadienoylcarnitine). Patients were categorized into four groups: group 1 consisted of patients with levels of both metabolites below their respective median values; group 2 included patients with 2-trans,4-cis-decadienoylcarnitine levels above its median and linoleamide levels below its median; group 3 comprised patients with linoleamide levels above its median and 2-trans,4-cis-decadienoylcarnitine levels below its median; and group 4 consisted of patients with levels of both metabolites above their respective medians. The different Kaplan-Meier curves for each group were compared globally using the Log-Rank test. A post-hoc analysis was subsequently performed, with p-values adjusted using the Bonferroni-Holm method to evaluate significant pairwise differences. Significancy threshold was set to p = 0.05. All statistical analyses were performed using SPSS software (version 20.0; IBM SPSS Inc; Chicago. IL, USA) and R commander program (version R-4.2.3) and RStudio (version 2023.3.0.386). The specific R packages employed were survival (version 3.6.4), survminer (version 0.4.9), ggsurvfit (version 1.1.0), and timeROC (version 0.4).

**EXTENDED DISCUSSION**

The incidence and prevalence of HFpEF continue to rise in tandem with the increasing age and burdens of obesity, sedentariness, and cardiometabolic disorders^1^. Despite major advances in pharmacological treatment for patients with HF, residual mortality remains high. This suggests that important pathways are not yet targeted by current HF therapies^2^. Taking into account these considerations, the discovery of plasma metabolites linked to HFpEF may contribute to a better knowlegde of its pathophysiology, clarify clinical phenotypes and prognosis, and perhaps aid in the development of targeted therapies for HFpEF patients. Initially, in the plasma of HFpEF patients compared to healthy CNT group we identified differential expression profiles of metabolites such as glutathione, which has a preventive role in cardiovascular diseases being able to respond to the increase in oxidizing agents^3^; sulfolithocholylglycine, a bile acid altered also in patients with hepatocellular carcinoma^4^ and lysophospholipids, that represented the largest subgroup of lipids found to be associated with incident myocardial infarction^5^.

Following this, we were interested in whether there were metabolomic alterations between HFpEF patients who suffered an event with those who did not. Here, we observed alterations in metabolites levels such as 5-amino-pentanoic acid, which is related to lysine degradation in cardiomyocyte hypertrophy^6^ or L-Urobilinogen that belongs to the heterogenous group of degradation products of bilirubin^7^. However, linoleamide presented the most altered levels, which were elevated in HFpEF patients who suffered an event. Linoleamide is an endogenous fatty acid primary amide, which is structurally related to sphingosine and sphiganine, and increases cytosolic Ca^2+^ levels in renal tubular cells^8^. Later, Yamamoto et al, described linoleamide as a modulator of intracellular Ca^2+^ homeostasis via regulation of SERCA activity^9^, and it has been also identified as a marker of patients with chronic lymphocytic leukaemia in its aggressive form^10^. Previously, circulating sphingosine-1-phosphate (S1P), a metabolite also closely related with SERCA, was suggested as a potential approach to detect cardiac rejection^11^. Furthermore, HF patients showed significant changes in key sphingolipid pathways and S1P accumulation^12^. However, there is limited understanding of the role for SERCA in HFpEF pathophysiology given the few human tissue sample studies and the lack of a comprehensive animal model of HFpEF^13^. Nonetheless, in a biopsy study of 20 patients with HFpEF and 11 patients with HFrEF, impaired calcium homeostasis was observed in both HF phenotypes, but reduced SERCA activity was noted only in diabetic patients with HFpEF^14^. On the other hand, it is widely known that the heart is a mitochondrion-rich tissue, and at the molecular level, impaired mitochondrial function was suggested to contribute to HFpEF development^15^. Circulating plasma acylcarnitines, which are intermediates of mitochondrial β-oxidation of fatty acids, are an emerging molecular signature of HF and are thought to reflect mitochondrial dysfunction^16-18^. Therefore, it is well-established that alterations in acylcarnitines levels are associated with HF risk^19-20^. We found that 2-trans,4-cis-decadienoylcarnitine, a medium-chain acylcarnitine, was also upregulated in HFpEF patients who experienced an event of death or readmission for cardiovascular causes compared with patients who did not. Roe et al observed that β-oxidation of linoleic acid yields 2-trans,4-cis-decadienoyl-CoA as an intermediate that, in the presence of NADPH, can be reduced by 2,4-dienoyl-CoA reductase and then further degraded by β-oxidation. If 2,4-dienoyl-CoA reductase is completely or partially missing, 2-trans,4-cis-decadienoyl-CoA would be expected to accumulate in mitochondria and be excreted as 2-trans,4-cis-decadienoylcarnitine^21^. Furthermore, the accumulation of 2-trans,4-cis-decadienoylcarnitine in plasma has been attributed to the dysfunction of mitochondrial NADP-dependent enzymes^22^. In line with previous findings, we observed increased levels of 2-trans,4-cis-decadienoylcarnitine, reinforcing its association with mitochondrial impairment in HFpEF patients. To our knowledge, this is the first study to provide new information about the role of linoleamide and 2-trans,4-cis-decadienoylcarnitine in HFpEF.

**REFERENCES**

1. Borlaug BA, Sharma K, Shah SJ, Ho JE. Heart Failure With Preserved Ejection Fraction: JACC Scientific Statement. *J Am Coll Cardiol*. 2023; 81(18):1810-1834. doi: 10.1016/j.jacc.2023.01.049

2. Ouwerkerk W, Belo Pereira JP, Maasland T, et al. Multiomics Analysis Provides Novel Pathways Related to Progression of Heart Failure. *J Am Coll Cardiol*. 2023; 82(20):1921-1931. doi: 10.1016/j.jacc.2023.08.053

3. Matuz-Mares D, Riveros-Rosas H, Vilchis-Landeros MM, Vázquez-Meza H. Glutathione Participation in the Prevention of Cardiovascular Diseases. *Antioxidants (Basel)*. 2021; 10(8):1220. doi:10.3390/antiox10081220

4. Li ZC, Wang J, Liu HB, et al. Proteomic and metabolomic features in patients with HCC responding to lenvatinib and anti-PD1 therapy. *Cell Rep*. 2024; 43(3):113877. doi: 10.1016/j.celrep.2024.113877

5. Nogal A, Alkis T, Lee Y, et al. Predictive metabolites for incident myocardial infarction: a two-step meta-analysis of individual patient data from six cohorts comprising 7897 individuals from the COnsortium of METabolomics Studies. *Cardiovasc Res*. 2023; 119(17):2743-2754. doi:10.1093/cvr/cvad147

6. Liu J, Hu J, Tan L, Zhou Q, Wu X. Abnormalities in lysine degradation are involved in early cardiomyocyte hypertrophy development in pressure-overloaded rats. *BMC Cardiovasc Disord*. 2021;21(1):403. doi:10.1186/s12872-021-02209-w

7. Vítek L, Majer F, Muchová L, et al. Identification of bilirubin reduction products formed by Clostridium perfringens isolated from human neonatal fecal flora. *J Chromatogr B Analyt Technol Biomed Life Sci*. 2006;833(2):149-157. doi:10.1016/j.jchromb.2006.01.032

8. Huang JK, Jan CR. Linoleamide, a brain lipid that induces sleep, increases cytosolic Ca2+ levels in MDCK renal tubular cells. *Life Sci*. 2001; 68(9):997-1004. doi:10.1016/s0024-3205(00)01002-x

9. Yamamoto S, Takehara M, Ushimaru M. Inhibitory action of linoleamide and oleamide toward sarco/endoplasmic reticulum Ca^2+^-ATPase. *Biochim Biophys Acta Gen Subj*. 2017; 1861 (1 Pt A):3399-3405. doi: 10.1016/j.bbagen.2016.09.001

10. Piszcz J, Armitage EG, Ferrarini A, et al. To treat or not to treat: metabolomics reveals biomarkers for treatment indication in chronic lymphocytic leukaemia patients. *Oncotarget*. 2016; 7(16):22324-22338. doi:10.18632/oncotarget.8078

11. Tarazón E, Gil-Cayuela C, Manzanares MG, et al. Circulating Sphingosine-1-Phosphate as A Non-Invasive Biomarker of Heart Transplant Rejection. *Sci Rep*. 2019; 9(1):13880. doi:10.1038/s41598-019-50413-8

12. Pérez-Carrillo L, Giménez-Escamilla I, Martínez-Dolz L, et al. Implication of Sphingolipid Metabolism Gene Dysregulation and Cardiac Sphingosine-1-Phosphate Accumulation in Heart Failure. *Biomedicines*. 2022; 10(1):135. doi:10.3390/biomedicines10010135

13. Sarma S, MacNamara JP, Hieda M, et al. SERCA2a Agonist Effects on Cardiac Performance During Exercise in Heart Failure With Preserved Ejection Fraction. *JACC Heart Fail*. 2023; 11(7):760-771. doi: 10.1016/j.jchf.2023.02.006

14. Frisk M, Le C, Shen X, et al. Etiology-Dependent Impairment of Diastolic Cardiomyocyte Calcium Homeostasis in Heart Failure With Preserved Ejection Fraction. *J Am Coll Cardiol*. 2021; 77(4):405-419. doi: 10.1016/j.jacc.2020.11.044

15. Kumar AA, Kelly DP, Chirinos JA. Mitochondrial Dysfunction in Heart Failure With Preserved Ejection Fraction. *Circulation*. 2019; 139(11):1435-1450. doi:10.1161/CIRCULATIONAHA.118.036259

16. Hunter WG, Kelly JP, McGarrah RW 3rd, Kraus WE, Shah SH. Metabolic Dysfunction in Heart Failure: Diagnostic, Prognostic, and Pathophysiologic Insights From Metabolomic Profiling. *Curr Heart Fail Rep*. 2016; 13(3):119-131. doi:10.1007/s11897-016-0289-5

17. Margulies KB, Hernandez AF, Redfield MM, et al. Effects of Liraglutide on Clinical Stability Among Patients With Advanced Heart Failure and Reduced Ejection Fraction: A Randomized Clinical Trial. *JAMA*. 2016; 316(5):500-508. doi:10.1001/jama.2016.10260

18. Hunter WG, Kelly JP, McGarrah RW 3rd, et al. Metabolomic Profiling Identifies Novel Circulating Biomarkers of Mitochondrial Dysfunction Differentially Elevated in Heart Failure With Preserved Versus Reduced Ejection Fraction: Evidence for Shared Metabolic Impairments in Clinical Heart Failure. *J Am Heart Assoc*. 2016;5(8): e003190. doi:10.1161/JAHA.115.003190

19. Ruiz-Canela M, Guasch-Ferré M, Razquin C, et al. Plasma acylcarnitines and risk of incident heart failure and atrial fibrillation: the Prevención con dieta mediterránea study. *Rev Esp Cardiol (Engl Ed)*. 2022; 75(8):649-658. doi: 10.1016/j.rec.2021.10.005

20. Ruiz M, Labarthe F, Fortier A, et al. Circulating acylcarnitine profile in human heart failure: a surrogate of fatty acid metabolic dysregulation in mitochondria and beyond. *Am J Physiol Heart Circ Physiol*. 2017;313(4):H768-H781. doi:10.1152/ajpheart.00820.2016

21. Roe CR, Millington DS, Norwood DL, Kodo N, Sprecher H, Mohammed BS, Nada M, Schulz H, McVieR. 2,4-Dienoyl-coenzyme A reductase deficiency: a possible new disorder of fatty acid oxidation. *J Clin Invest*. 1990; 85(5):1703-1707. doi:10.1172/JCI114624

22. Zhang R, Zhang K. Mitochondrial NAD kinase in health and disease. *Redox Biol*. 2023; 60:102613. doi: 10.1016/j.redox.2023.102613
